# Neuroprotective Effect of Polyherbal Recipe Containing Ginger, Chinese Date, and Wood Ear Mushroom against Ischemic Stroke with Metabolic Syndrome Condition via Epigenetic Modification of Inflammation and Oxidative Stress

**DOI:** 10.1155/2022/8940303

**Published:** 2022-08-17

**Authors:** Thuntiva Nakyam, Jintanaporn Wattanathorn, Wipawee Thukham-mee

**Affiliations:** ^1^Department of Physiology and Graduate School (Neuroscience Program), Faculty of Medicine, Khon Kaen University, Khon Kaen, Thailand 40002; ^2^Research Institute for Human High Performance and Health Promotion, Khon Kaen University, Khon Kaen, Thailand 40002; ^3^Department of Physiology, Faculty of Medicine, Khon Kaen University, Khon Kaen, Thailand 40002

## Abstract

Currently, the prevalence of stroke with metabolic syndrome (MetS) is increasing and the current therapeutic efficiency is still limited. Therefore, the applications of herbal recipes have gained much attention. The polyherbal recipe containing ginger, Chinese date, and wood ear mushroom is reputed for atherosclerosis and stroke prevention. It has been long-term consumed without scientific support. Therefore, this study was carried out to determine the neuroprotective effect and its mechanisms in animal model of ischemic stroke with MetS. Male Wistar rats weighing 180-220 g were exposed to a 16-week high-fat high-carbohydrate feeding. The rats with the MetS characteristic were exposed to a temporary occlusion of the right middle cerebral artery (MCAO) for 90 minutes. They were orally fed with the polyherbal recipe (GCJ) at the doses of 100, 200, and 300 mg/kg BW for 21 days and assessed the neurological deficit, ion volume, cortical neuron density in the cerebral cortex, oxidative stress status, inflammation, and expressions of histone deacetylase 3 (HDAC3) and DNA methyltransferase 1 (DNMT1). The results showed that GCJ significantly improved all mentioned parameters. Therefore, GCJ is the potential neuroprotectant against ischemic stroke with MetS. The underlying mechanisms may involve the reduction of oxidative stress, inflammation, and the modification of epigenetic mechanism via the reduction of HDAC3 and DNMT1. However, further clinical investigation is essential to confirm this positive modulation effect.

## 1. Introduction

Metabolic syndrome (Mets), a cluster of conditions including hypertension, diabetes, atherogenic dyslipidemia, and central obesity, is recognized as an important risk factor for stroke development [1, 2]. MetS disturbs cerebral microvascular blood flow giving rise to the reduction of cerebral blood flow and finally exposing the brain tissue to a higher state of hypoxia [3]. In addition, the elevation of prooxidative and proinflammatory states induced by MetS also contributes an important role on the development of atherosclerotic plaque and thrombosis-embolic stroke [4]. Therefore, the prevalence of stroke with MetS appears to increase in accompany with the increasing trend of MetS [5]. It has been reported that stroke with MetS shows poor prognosis. In addition, stroke survivors with MetS also show the higher mortality rate and stroke recurrence than those without MetS [6]. Since stroke produces a great socioeconomic burden and the current therapeutic strategy is still limited, it is very essential to prevent it [7].

At present, the application of herbal products for stroke prevention has gained much attention. Although the herbal products in the market are present both in single herb formulation and in polyherbal formulation, the concept of polyherbalism has gained much attention because the active phytochemical constituents presented in each individual plant usually exist in a small amount and do not achieve therapeutic levels. The combination of herbs often provides greater benefits due to the synergistic interaction. One possible explanation for the positive modulation effect induced by synergistic effect is the capacity to exert the effect at multiple targets simultaneously which is suitable for the complex disorders which involve multiple factors. In addition, most polyherbal formulation often provides the desirable effect with lower dose than the single formulation [8]. In Thailand, the polyherbal drink containing ginger (*Zingiber officinale* Roscoe), Chinese date (*Ziziphus jujuba* Mill.), and wood ear mushroom or Jew's ear mushroom (*Auricularia auricula-judae*) has been consumed by the Thai-Chinese descents in the northern part of Thailand based on the belief that this polyherbal drink can improve blood cholesterol and atherosclerosis. Due to the crucial role of atherosclerosis on the pathophysiology of stroke [2], the polyherbal drink mentioned earlier is also used for preventing ischemic stroke. However, no scientific evidence concerning this effect is available. Therefore, this study was set up to determine the effect of this polyherbal recipe and to explore its underlying mechanisms in animal model of metabolic syndrome with ischemic stroke.

## 2. Materials and Methods

### 2.1. Preparation of the Polyherbal Recipe (GCJ)

Ginger, Chinese date, and wood ear mushroom were harvested from October 2020 to January 2021. The ginger (*Zingiber officinale* Roscoe) was authenticated by the KKU herbarium (KKU-NO. 25652), and Chinese date (*Ziziphus jujube* Mill.) was identified by KKU herbarium (KKU-NO. 25654). The voucher specimens ginger and Chinese date were authenticated by Assistant Professor Pimwadee Pornpongrungrueng, the plant taxonomy expert from the Faculty of Science, Khon Kaen University. The wood ear or the Jew's ear mushroom (*Auricularia auricula-judae* Bull.) utilized for this study was sourced from Mahasarakham province, International Herbarium Index (MSUT-7282). Voucher specimens were authenticated by Assistant Professor Khwanruan Naksuwankul and deposited at High Human Performance and Health Promotion Research Institute (HHP&HP), Khon Kaen University, Thailand. For the extract preparation, ginger, Chinese date, and wood ear mushroom were washed, dried in the oven (72 hours at 60°C), and grounded as powder. Both ginger and wood ear mushroom were prepared as water extract by using a decoction technique whereas a water extract of Chinese date was prepared using maceration technique. All extracts were centrifuged at 3000 rpm for 10 minutes. Following this process, the solution was harvested and filtrated with Whatman paper. Then, they were dried with a freeze dryer. The polyherbal recipe was prepared by combining all extracts at a ratio of 1 : 1 : 1 owing to the highest potential in biological activities associated with the pathophysiology of metabolic syndrome and ischemic stroke.

### 2.2. Experimental Protocol

All animals used in this study were male Wistar rats weighing 180-220 g from Nomura Siam International Co., Ltd., Thailand. They were housed in standard metal cages (6 animals per cage), under the standard condition with 23 ± 2°C, 12 : 12 hours of the light-dark cycle, and were allowed free access to water and food throughout the study. All animals were subjected to a one-week acclimatization period. All procedures in this study were approved by the Institutional Animal Ethics Committee (report no. IACUC-KKU 42/63).

**Figure 1:**
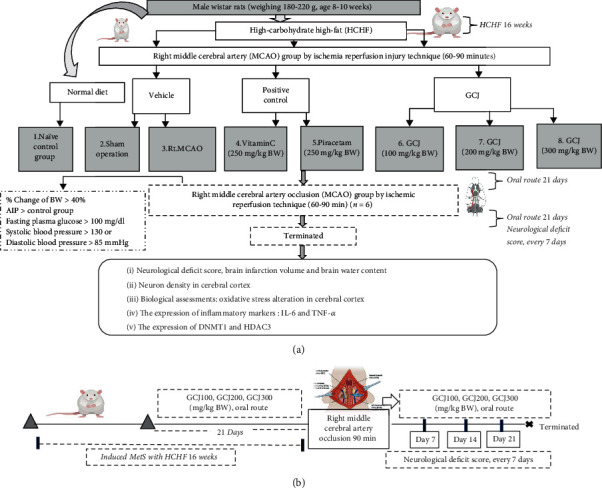
Schematic diagram showing all interventions and assessments used in this study. (a) Diagram of the experimental protocol showing all interventions and assessments. (b) Time schedules of treatments, the occlusion of middle cerebral artery, and the neurological deficit assessment.

After acclimatization, all rats were randomly divided into 8 groups (*N* = 6/group). Rats in group I (ND+vehicle) were treated with a normal diet (4.5% fat, 44.8% carbohydrate, 24% protein, and 5% fiber) whereas rats in group II-group VIII were fed with high-carbohydrate high-fat diet (HCHF) (19.36% fat, 52.94% carbohydrate, 22.2% protein, and 3.28% fiber) in order to induce metabolic syndrome and this rat model can produce the physiological changes and manifestation mimic metabolic syndrome in human better than other models [9, 10]. To mimic the real situation in MetS, only rats that showed the characteristic similar to those presented in the human metabolic syndrome condition such as obesity (the body weight gain more than 40% [11]), hyperglycemia (the plasma glucose more than 100 mg/dL [12]), and hypertension (systolic and diastolic pressure more than 130 and 85 mmHg [13]) were recruited for further study. Rats in group II (HCHF+sham+vehicle) were exposed to sham operation while rats in group III-group VIII were exposed to a temporary occlusion of the right middle cerebral artery (MCAO) via a 90-minute reperfusion injury. Group II was designed to observe the effect of sham operation and vehicle on the interested brain parameters. After MCAO, rats in group III (HCHF+MCAO+vehicle), group IV (HCHF+MCAO+vitamin C), and group V (HCHF+MCAO+piracetam) were fed with vehicle, vitamin C (250 mg/kg BW), and piracetam (250 mg/kg BW), respectively. Group III was designed to observe the effect of MCAO which mimicked brain injury, and brain dysfunction presented in ischemic stroke because vehicle is water and produced no effect on the parameters just mentioned. Both group IV and group V were served as the positive control group because both vitamin C (a well-known antioxidant) and piracetam (a standard drug used for treating stroke in order to improve cerebral blood flow and neuroinflammation) showed the positive modulation effects on the observed parameters and they were included for comparing the observed effects with the experimental group. In groups VI-VIII (HCHF+MCAO+GCJ), all MetS rats were fed with GCJ at the doses of 100, 200, and 300 mg/kg BW, respectively. These groups were experimental groups. The administrations of the assigned substances were performed continually for 21 days. The assessment of severity of neurological deficit was carried out by using modified neurological severity scores (mNSS) every 7 days throughout the study period. The determination of brain infarction volume, brain edema, brain oxidative stress markers including malondialdehyde (MDA) level, and the activities of catalase (CAT), glutathione peroxidase (GPx), and superoxide dismutase (SOD) together with the assessment of inflammatory markers such as tumor necrosis factor-alpha (TNF-*α*) and interleukin-6 (IL-6) was also performed. In addition, the modification of epigenetic mechanism was also evaluated via the changes in histone deacetylase 3 (HDAC3) and DNA methyltransferase 1 (DNMT1) expressions. All experimental protocols are shown in Figure 1.

### 2.3. Ischemia-Reperfusion (I/R) Injury

After an anesthetization with pentobarbital sodium (60 mg/kg BW), a midline incision of the neck was performed. Following this step, a silicon-coated nylon was inserted into the right common carotid artery lumen and passed through the internal carotid artery until the middle cerebral artery was occluded. The occlusion was performed for 90 minutes, and the nylon was removed to induce reperfusion injury. Sham-operated rats were subjected to the same processes except that no occlusion of a middle cerebral artery was performed. After the operation, the wound was sutured, and an animal was transferred to the cage after recovering from an anesthetization [14–16].

### 2.4. The Modified Method of Neurological Severity Score (mNSS) Assessment

The neurological deficits comprising motor, sensory, reflex, and balance were monitored by using the modified method of neurological severity scores (mNSS). The neurological deficit score was graded by the trained and experienced observers who were blind to the treatment groups of the animals. The scores were in the range between 0 and 18. The mild injury represented the score between 1 and 6 whereas moderate injury represented the score between 7 and 12 and the scores in the range between 13 and 18 indicated severe injury [17, 18].

### 2.5. Assessment of Brain Infarction Volume

An assessment was performed 24 hours after MCAO due to the optimum change of brain infarction induced by MCAO during this period [19]. Brain was isolated and cut as a coronal section at 2 mm thickness. Each section was immersed in 2.0% 2,3,5-TTC (Sigma, St. Louis, MO, USA) for 30 min at 37°C (15 min with each surface facing up). All slices were washed twice in saline, fixed in 10% buffered formalin (pH 7.4), and left to stand at 4°C overnight in a lightproof container [15, 16].

### 2.6. Brain Water Content Assessment

The brain tissue collection was performed at 24 hours after a reperfusion injury. An assessment was carried out by weighing the brain wet weight and then subsequently allowed to dry at 100°C for 24 hours. Following this step, it was measured as brain dry weight. The final brain water content was calculated using [14]

(1) $$\begin{array}{c} \text{Water content}=\frac{\left(\text{wet weight}-\text{dry weight}\right)}{\text{wet weight}}\times 100\%. \end{array}$$

### 2.7. Cresyl Violet Staining

The isolated brains were fixed with 4% of paraformaldehyde (Sigma-Aldrich, USA) (pH 7.4) at 4°C and cryoprotected by immersing in a 30% formalin-sucrose (Merck, Germany) solution for 2-3 days. Then, they were prepared as a coronal section at 10 *μ*m thick by using cryostat (Thermo Scientific™ HM 525 Cryostat). All sections were placed on slides coated with 0.3 percent gelatin buffer containing 0.05% aluminium potassium sulfate (Sigma-Aldrich, USA). Prior to the cresyl violet staining process, all slides were air dried and rehydrated in graded concentrations of ethanol and xylene, respectively. Then, they were incubated in 0.2% of cresyl violet (Sigma-Aldrich, USA) for 20 minutes accordingly [20, 21], decolorized in acetic acid, dehydrated, and cover slipped with Permount. The evaluation of neuronal density in the sensorimotor cortex (bregma 1.6 to -3.0, anterior to posterior) was performed by using an Olympus light microscope type BH-2 (Japan) at 40x magnification. The analysis was performed specifically focused on neuropil, neuronal size, and shape [22]. The density of the survival neurons was expressed as the number of neurons per 255 *μ*m^2^ [22].

### 2.8. Oxidative Stress Marker Assessment

#### 2.8.1. Lipid Peroxidation Assessment

Level of malondialdehyde (MDA) in the cerebral cortex was monitored by using thiobarbituric acid reactive substances (TBARSs) method [23]. Briefly, 50 *μ*L of tissue homogenate, 50 *μ*L sodium dodecyl sulfate solution (SDS; Sigma-Aldrich, USA), 375 *μ*L 20% acetic acid solution, 375 *μ*L of 0.8% of thiobarbituric acid (TBA) (Sigma-Aldrich, USA), and 150 *μ*L of distilled water (DW) were mixed and heated in 95°C for 1 hour. After the samples were cooled, 250 *μ*L of distilled water and 1250 *μ*L of mixture of n-butanol : pyridine (15 : 1 *v*/*v*; Merck, Germany) were added and centrifuged for 10 minutes at 4000 rpm to extract the TBARS (pink complex color). Butanolic phase was collected and measured an absorbance at 532 nm. The brain level of MDA was calculated by using a 1,3,3-tetramethoxy propane (Sigma-Aldrich 108383-100ML) standard curve at the concentration range between 0 and 15 *μ*M. The measurement was performed in triplicate, and results were expressed as ng/mg protein.

#### 2.8.2. Assessment of Antioxidant Enzymes

Superoxide dismutase activity (SOD) was monitored via the presence of superoxide free radical generated by the cytochrome C reduction [24]. In brief, 200 *μ*L of the reaction mixture comprising 57 mM of phosphate buffer solution (KH_2_PO_4_), 0.1 mM ethylenediaminetetraacetic acid (EDTA) (Sigma-Aldrich, USA), 50 *μ*M of xanthine solution, and 10 mM of cytochrome C solution (Sigma-Aldrich, USA) was mixed with 20 *μ*L of samples or 1 to 25 units/mL of superoxide dismutase enzyme standard (Sigma-Aldrich, USA). An absorbance was assessed at a wavelength of 415 nm. The unit of measurement in SOD activity was expressed as SOD activity per mg protein.

Catalase (CAT) activity was determined following the method of Palachai et al. [25]. The assay mixture consisting of 50 *μ*L of 30 mM of H_2_O_2_ in 50 mM phosphate buffer, pH 7.0, 150 *μ*L of 5 mM KMnO_4_, and 25 *μ*L of H_2_SO_4_ (Sigma-Aldrich, USA) was mixed with 10 *μ*L of brain homogenate and determined an absorbance at 490 nm by using a microplate reader. An enzymatic assessment was performed in triplicate, and results were expressed as units of CAT activity per milligram of protein (U/mg protein).

The determination in glutathione peroxidase (GPx) activity was performed by adding 20 *μ*L of tissue sample to the reaction mixture containing 10 *μ*L of 1 mM dithiothreitol (DTT) in 6.67 mM potassium phosphate buffer (pH 7), 100 *μ*L of 1 mM sodium azide (Sigma-Aldrich, USA), 10 *μ*L of 50 mM glutathione solution, and 100 *μ*L of 30% H_2_O_2_ (BDH Chemicals Ltd., UK). Subsequently, 10 *μ*L of 10 mM DTNB (5,5-dithiobis-2-nitrobenzoic acid) (Sigma-Aldrich, USA) was added and incubated for 5 minutes at room temperature. An absorbance was assessed using a microplate reader at a wavelength of 412 nm. Results were presented as units per mg protein [25].

### 2.9. Western Blot Assessment

Inflammation and epigenetic mechanism play the crucial roles in brain damage induced by ischemic stroke [22, 26–28], so we also determined the effect of the polyherbal recipe on these indicators. In this study, we used western blot for determining the expressions of inflammatory cytokines such as interlukin-6 (IL-6) and tumor necrosis factor-*α* (TNF-*α*) and the expressions of both histone deacetylase 3 (HDAC3) and DNA methyltransferase 1 (DNMT1) in the cerebral cortex. Brain samples of the cerebral cortex from an ipsilateral side were homogenized with the extraction of mammalian reagent (M-PER) and the protease cocktail inhibitor (Sigma-Aldrich, USA) at the ratio of 1 : 10 for the assessment of inflammatory markers. The brain samples were lysed in RIPA buffer or subjected to a radioimmunoprecipitation assay that incorporated 20 mM Tris-HCl pH 7.5, 150 mM NaCl, 1 mM EGTA, 1 mM Na_2_EDTA, 1% sodium deoxycholate, 1% NP-40, 1 mM beta-glycerophosphate, 1 mM Na_3_VO_4_, 2.5 mM sodium pyrophosphate, 1 *μ*g/mL leupeptin, and 1 mM phenylmethanesulfonyl fluoride for the assessment of HDAC3 and DNMT1. All brain homogenates were subjected to a 12,000*g* centrifugation at 4°C for 10 minutes. A volume of 80 *μ*g of sample was incubated with loading buffer Tris-Glycine SDS-PAGE (Bio-Rad, USA) for 10 minutes at 95°C. Then, 20 *μ*L of the mixture was loaded on SDS-polyacrylamide gel and separated it by sodium dodecyl sulfate-polyacrylamide gel electrophoresis (SDS-PAGE). Following the electrophoresis process, protein bands were transferred from the gel to nitrocellulose membranes, and they were washed with 0.05% TBS-T. After the washing process, the membranes were incubated in a blocking buffer containing 5% skim milk and 0.1% TBS-T at 25°C for 1 hour. At the end of an incubation period, they were incubated with one of the following primary antibodies: IL-6 (dilution 1 : 1000), TNF-*α* (dilution 1 : 1000), DNMT-1 (dilution 1 : 1000), HDAC-3 (dilution 1 : 1000), and *β*-actin (dilution 1 : 2000) (Cell Signaling Technology, USA) for 2 hours at 25°C. All membranes were then washed with 0.05% T-PBS for 30 minutes. Further incubation with secondary enzyme-linked antibody (anti-rabbit IgG, HRP-linked antibody obtained from Cell Signaling Technology, USA; dilution 1 : 2000) was carried out to enhance signal transduction. The protein band densities were detected using ECL detection systems and the LAS-4000 luminescent image analyzer (GE Healthcare). ImageJ software system was used for the analysis of the band densities. The band density control normal group's numeric variables were expressed as relative density [25].

### 2.10. Statistical Analysis

Data obtained from this study were expressed as mean ± SEM and statistically analyzed using SPSS software version 25.0. The statistical difference between the groups was obtained using a one-way analysis of variance (ANOVA) whereas the data analyzed repetitively were performed with repeated measurement ANOVA, followed by a Tukey post hoc test. The *p* values below 0.05 were considered as significant changes between the test groups.

## 3. Results

### 3.1. The Modified Neurological Severity Score (mNSS), Brain Infarction Volume, and Brain Water Content

**Figure 2:**
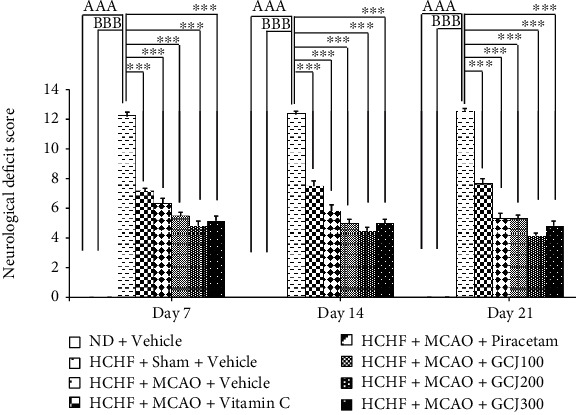
Effect of the polyherbal recipe containing ginger, Chinese date, and wood ear mushroom (GCJ) at the doses of 100, 200, and 300 mg/kg BW on the neurological deficit score assessed using the method of neurological severity scores (mNSS). Data were presented as mean ± SEM (*N* = 6/group). ^AAA^Significant difference from the naïve control group (ND+vehicle) at *p* value < 0.001; ^BBB^significant difference from HCHF diet treated rats plus sham operation group (HCHF+sham+vehicle); ^∗∗∗^significant difference from HCHF diet treated rats plus MCAO and vehicle (HCHF+MCAO+vehicle).

Figure 2 shows the motor deficit assessed by using mNSS. Our results showed that HCHF diet treated rats which received sham operation showed no significant neurological deficit whereas HCHF diet treated rats which received MCAO showed the significant increase in mNSS when compared to the rats which received normal diet and vehicle treatments throughout the study period (*p* value < 0.001, all). These data suggested that HCHF plus sham operation and vehicle treatment produced no influence on mNSS and the alteration in mNSS observed in HCHF+MCAO+vehicle appeared to occur as the result of MCAO. When compared between HCHF diet treated rats subjected to sham operation and HCHF diet treated rats which subjected to MCAO, our data showed that MCAO significantly increased mNSS in HCHF diet treated rats at 7, 14, and 21 days after MCAO (*p* value < 0.001, all). These data pointed out that MCAO played an essential role on the elevation of mNSS. The significant elevation in mNSS induced by MCAO was mitigated by vitamin C, piracetam, and GCJ at the doses of 100, 200, and 300 mg/kg BW throughout the study period (*p* value < 0.001, all) as shown in Figure 2. Owing to the same pattern of changes in mNSS induced by the substances mentioned earlier, the similar underlying mechanisms of the tested substances had been considered.

**Figure 3:**
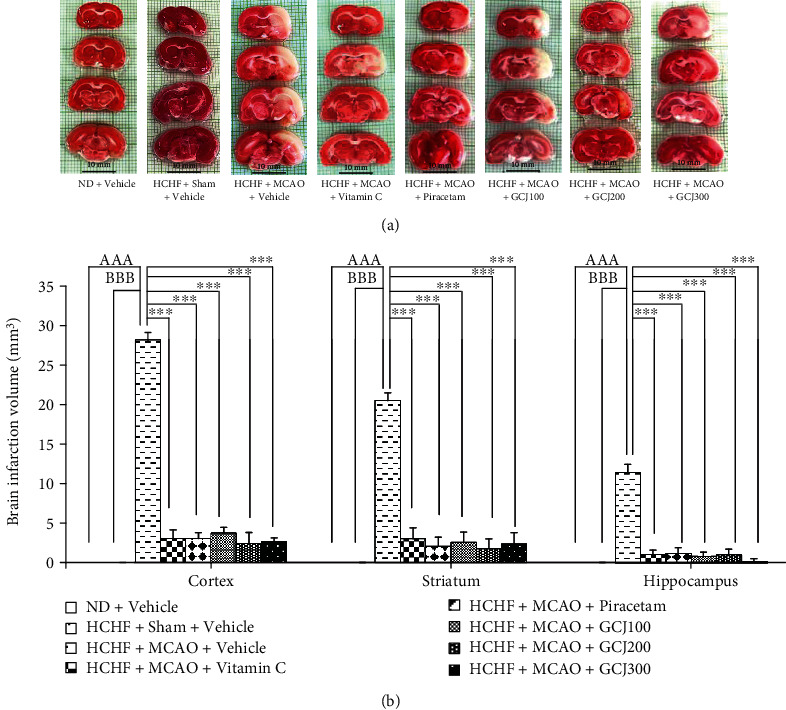
Effect of the polyherbal recipe containing ginger, Chinese date, and wood ear mushroom (GCJ) at the doses of 100, 200, and 300 mg/kg BW on brain infarction volume. (a) Representative TTC staining image of all treatment groups. (b) Brain infarction area in the cerebral cortex, striatum, and hippocampus of all treatment groups. Data were expressed as mean ± SEM (*N* = 6/group). ^AAA^*p* value < 0.001 vs. ND+vehicle; ^BBB^*p* value < 0.001 vs. HCHF+sham+vehicle; ^∗∗∗^*p* value < 0.001 vs. HCHF+MCAO+vehicle.

The effect of various treatments in this study on brain infarction volume was also monitored, and results are shown in Figure 3. It was found that no brain infarction volume was observed in rats fed with normal diet and received vehicle (ND+vehicle) and in rats fed with HCHF diet and received sham operation plus vehicle treatment. These data suggested that HCHF plus sham operation and vehicle treatment failed to produce brain infarction. MCAO significantly increased brain infarction volume in the cerebral cortex, striatum, and hippocampus when compared to the ND+vehicle group (*p* value < 0.001). The comparison of brain infarction volume between HCHF+MCAO+vehicle treated rats and HCHF+sham+vehicle treated also showed the same pattern (*p* value < 0.001, all). Therefore, a brain infarction volume observed in this group occurred because of MCAO. All treatments in this study including the treatments with vitamin C, piracetam, and all doses of GCJ used in this study showed the significant mitigation in brain infarction volume in all brain regions mentioned earlier of HCHF diet treated rats subjected to MCAO (*p* value < 0.001, all). These data also pointed out that the mitigation effect on brain infarction of the GCJ mentioned earlier might occur via the reduction of oxidative stress and the improvement of cerebral blood flow similar to the effects of vitamin C and piracetam.

**Figure 4:**
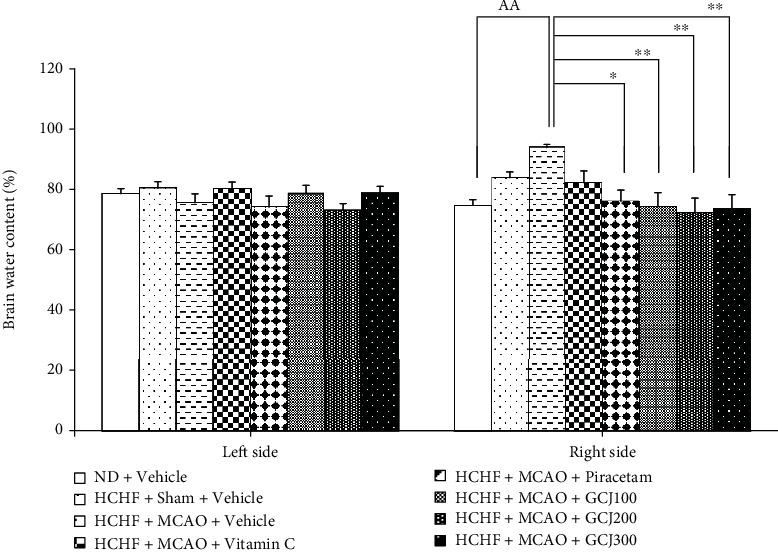
Effect of the polyherbal recipe containing ginger, Chinese date, and wood ear mushroom (GCJ) at the doses of 100, 200, and 300 mg/kg BW on both contralateral (left) and ipsilateral (right) brain water content. Data were as expressed as mean ± SEM (*N* = 6/group). ^AA^*p* value < 0.01, compared to ND+vehicle; ^∗,∗∗^*p* value < 0.05 and 0.001, respectively, compared to HCHF+MCAO+vehicle.

The effect of various interventions on brain water content is shown in Figure 4. The current data showed no significant difference in the contralateral brain water content. In addition, HCHF diet treated rats which received sham operation and vehicle also failed to show the significant change in the ipsilateral brain water content. These data indicated that HCHF, sham operation, and vehicle exerted no significant influence on the brain water content. When compared to ND+vehicle treated rats, MCAO significantly enhanced the ipsilateral brain water content (*p* value < 0.01) but no significant difference was observed when compared to HCHF+sham+vehicle. Therefore, HCHF+sham+vehicle might also produce slight change on brain edema although the change was not significant. This might be associated with the injury induced by an operation. Piracetam and GCJ at the doses of 100, 200, and 300 mg/kg BW significantly decreased the elevation of the ipsilateral brain water content in HCHF rats which received MCAO (*p* value < 0.05, 0.01, 0.01, and 0.01, respectively; compared to HCHF+MCAO+vehicle). These changes suggested that the improvement in brain edema was not principally attributed to an oxidative stress but might be principally associated with the alteration in the blood flow dynamic like the action of piracetam.

### 3.2. Cortical Neuron Density

**Figure 5:**
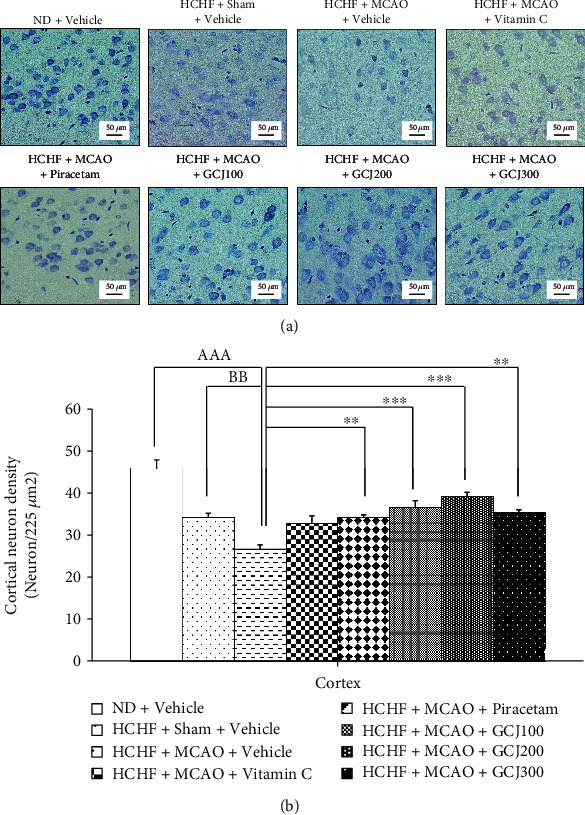
Effect of the polyherbal recipe containing ginger, Chinese date, and wood ear mushroom (GCJ) at the doses of 100, 200, and 300 mg/kg BW on cortical neuron density. (a) Light microscope of coronal sections in the cerebral cortex was stained with cresyl violet at 40x magnification. (b) Density of survival neurons in the cerebral cortex. Data were expressed as mean ± SEM (*N* = 6/group). ^AAA^*p* value < 0.001, compared to ND+vehicle; ^BB^*p* value < 0.01, compared to HCHF+sham+vehicle; ^∗∗,∗∗∗^*p* value < 0.01 and 0.001, respectively, compared to HCHF+MCAO+vehicle.

The effect of various interventions on cortical neuron density is shown in Figure 5. When compared to the ND+vehicle group, HCHF plus sham operation and vehicle treated rats showed a slight reduction in neuron density in the cerebral cortex but no significance was observed. MCAO significantly decreased the cortical neuron density in HCHF plus vehicle treated rats (*p* value < 0.001, compared to the ND+vehicle group; *p* value < 0.01, compared to the HCHF+sham+vehicle group). The reduction of neuron density in the cortical area induced by MCAO was attenuated by piracetam and GCJ at the doses of 100, 200, and 300 mg/kg BW (*p* value < 0.01, 0.001, 0.001, and 0.01, respectively; compared to HCHF+MCAO+vehicle treated rats). Therefore, GCJ at all dosage range used in this study might exert the neuroprotective effect via an antioxidant activity same as piracetam.

### 3.3. Oxidative Stress Changes

The effect of GCJ on the alteration of oxidative stress status of the cerebral cortex, a brain region which showed the optimum changes induced by MCAO, was monitored via the alterations of the level of a lipid peroxidation product or MDA level and the alterations of main antioxidant enzymes in the brain including SOD, CAT, and GPx.  Table 1 shows that HCHF+sham+vehicle treated rats failed to show the significant changes of all parameters mentioned above in the cerebral cortex. These data suggested that MetS rats induced by HCHF diet subjected to sham operation and vehicle treatment did not produce any significant effect on oxidative stress status. HCHF+MCAO+vehicle treated rats significantly decreased the activities of SOD (*p* value < 0.001, compared to ND+vehicle; *p* value < 0.001, compared to HCHF+sham+vehicle), CAT (*p* value < 0.001, compared to ND+vehicle; *p* value < 0.001, compared to HCHF+sham+vehicle), and GPx (*p* value < 0.01, compared to ND+vehicle). However, the level of MDA in this brain region of HCHF+MCAO+vehicle treated rats was increased (*p* value < 0.01, compared to ND+vehicle; *p* value < 0.01, compared to HCHF+sham+vehicle). Vitamin C and GCJ at all doses used in this study significantly mitigated the changes of MDA (*p* value < 0.05, 0.01, 0.01, and 0.1, respectively; compared to HCHF+MCAO+vehicle), SOD (*p* value < 0.001, all; compared to HCHF+MCAO+vehicle), and CAT (*p* value < 0.01, 0.01, 0.001, and 0.01, respectively; compared to HCHF+MCAO+vehicle). The significant increase in GPx was also observed in HCHF and MCAO treated rats which received vitamin C and GCJ at the doses of 200 and 300 mg/kg BW (*p* value < 0.05, all; compared to HCHF+MCAO+vehicle). Piracetam treatment could enhance only CAT activity in HCHF+MCAO treated rats (*p* value < 0.01, compared to HCHF+MCAO+vehicle).

### 3.4. Inflammation and Epigenetic Mechanism

**Figure 6:**
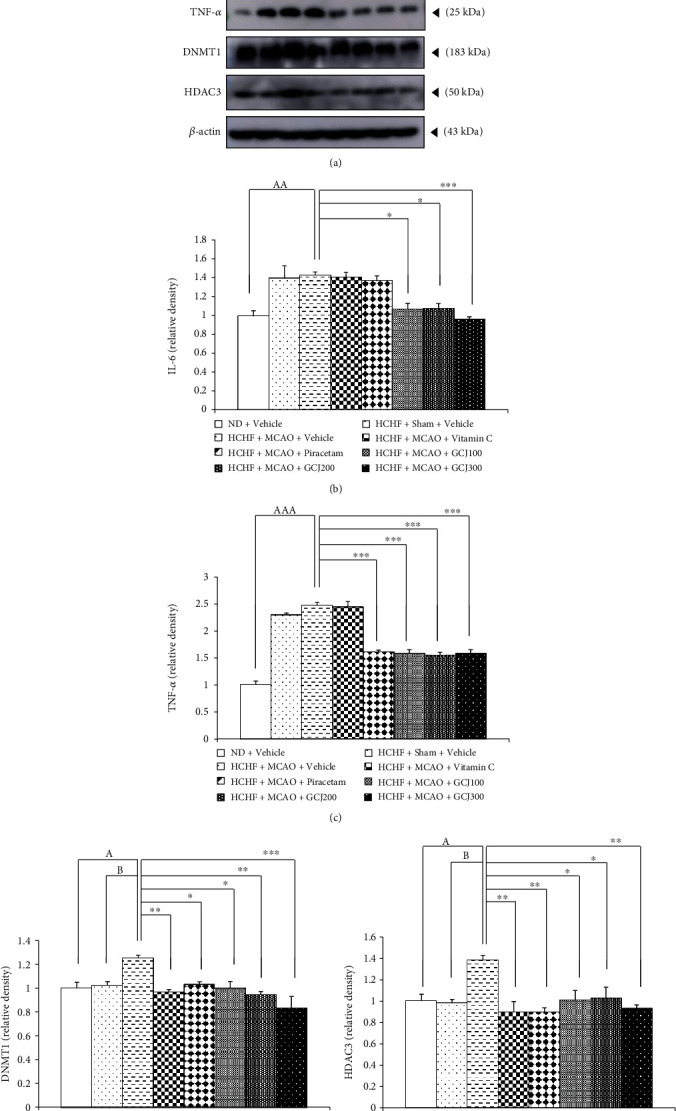
Effects of the polyherbal recipe containing ginger, Chinese date, and wood ear mushroom (GCJ) at the doses of 100, 200, and 300 mg/kg BW on the cortical expressions of interleukin-6 (IL-6) and tumor necrosis factor-*α* (TNF-*α*). (a) Representative bands of IL-6, TNF-*α*, DNMT1, and HDAC3 from the cerebral cortex of various treatment groups. Statistical data from the western blot analysis for the cortical expression of (b) IL-6, (c) TNF-*α*, (d) DNMT1, and (e) HDAC3. Data were expressed as mean ± SEM (*N* = 6/group). ^A,AA,AAA^*p* value < 0.05, 0.01, and 0.001, respectively, compared to ND+vehicle; ^B^*p* value < 0.05, compared to HCHF+sham+vehicle; ^∗,∗∗,∗∗∗^*p* value < 0.05, 0.01, and 0.001, respectively, compared to HCHF+MCAO+vehicle.

The effects of GCJ on the expressions of inflammatory cytokines such as IL-6 and TNF-*α* and epigenetic mechanism via the modification of HDAC3 and DNMT1 were also determined, and results are shown in Figure 6. Figures 6(a)–6(c) revealed that no significant changes in the cortical expressions of both IL-6 and TNF-*α* were observed in HCHF+sham+vehicle treated rats. Therefore, sham operation and vehicle treatment produced no effects on both HDAC3 and DNMT1 in MetS rats induced by HCHF. MCAO significantly increased the cortical expressions of IL-6 and TNF-*α* (*p* value < 0.01 and 0.001, respectively; compared to ND+vehicle) in MetS rats induced by HCHF diet. Interestingly, GCJ at the doses of 100, 200, and 300 mg/kg BW significantly decreased the expressions of IL-6 (*p* value < 0.05, 0.05, and 0.001, respectively; compared to HCHF+MCAO+vehicle) and TNF-*α* (*p* value < 0.001, all; compared to HCHF+MCAO+vehicle) while vitamin C failed to show the positive modulation effect on both IL-6 and TNF-*α* and piracetam significantly decreased only TNF-*α* expression (*p* value < 0.001, compared to HCHF+MCAO+vehicle).

Our data in Figures 6(a), 6(d), and 6(e) clearly demonstrated that HCHF+sham+vehicle treated rats failed to show the significant changes in both DNMT1 and HDAC3. These data suggested that sham operation did not modify the expressions of both parameters in MetS rats induced by HCHF diet. HCHF+MCAO+vehicle produced the significant elevation in the cortical expressions of the aforementioned parameters (*p* value <0.05, all, compared to HCHF+MCAO+vehicle and *p* value < 0.05, all, compared to HCHF+MCAO+vehicle). Therefore, the changes in DNMT1 and HDAC3 expression in MetS rats induced by HCHF diet might occur as the result of MCAO. The elevation of DNMT1 expression in the cerebral cortex of HCHF+MCAO+vehicle treated rats was attenuated by vitamin C, piracetam, and all doses of GCJ used in this study (*p* value < 0.01, 0.05, 0.05, 0.01, and 0.001, respectively; compared to HCHF+MCAO+vehicle). In addition, all interventions mentioned earlier also mitigated the elevation of HDAC3 in the cerebral cortex of HCHF+MCAO+vehicle treated rats (*p* value < 0.01, 0.01, 0.05, 0.05, and 0.001, respectively; compared to HCHF+MCAO+vehicle).

## 4. Discussion

This is the first report to demonstrate the neuroprotection against cerebral ischemia with metabolic syndrome of the reputed polyherbal recipe widely used in the Thai-Chinese descendants for a long time. Interestingly, it can exert the effect to modulate epigenetic mechanism via the suppression of DNMT1 and HDAC3 expression in the cerebral cortex. The changes are observed accompanied with the reduction of MDA, a lipid peroxidation marker, and inflammatory cytokines such as IL-6 and TNF-*α*, the elevation of antioxidant enzymes such as SOD, CAT, and GPx, and the reduction of brain infarction volume, neurodegeneration, brain edema, and neurological deficit.

In this study, a transient ischemic rat has been used as an animal model owing to its reputation as the best model for studying the survival and the functional recovery of ischemic stroke [29]. It has been demonstrated that a transient occlusion of a middle cerebral artery induces oxidative stress and neuroinflammation [30]. The substances possessing the suppression effect on oxidative stress and neuroinflammation such as polyphenol can improve brain damage and brain dysfunction in an ischemic stroke model [31, 32]. These pieces of information correspond with our results which demonstrated that all doses of GCJ, a polyphenol-enriched polyherbal recipe containing ginger, Chinese date, and wood ear mushroom, also improve both oxidative stress status and inflammation leading to an improvement of neurological deficit.

Recently, the role of epigenetic mechanism in the regulation of gene transcription in stroke has been clearly revealed [26, 33]. The current data showed the elevation of DNMT1 and HDAC3 in cerebral ischemia corresponding with the changes in the previous studies [34–39]. These changes are associated with brain edema [40], neuronal loss, neuronal deficit, and neuronal recovery in cerebral ischemia [41]. In addition, an epigenetic mechanism also plays an essential role on the regulation of inflammation [42–44]. Moreover, epigenetic mechanism process of the histone modification via HDAC3 also regulates oxidative stress balance. The suppression of HDAC3 can improve oxidative stress imbalance and brain damage in cerebral ischemia [25, 33]. The improvement in oxidative stress imbalance and inflammation in turn give rise to the decrease in neurodegeneration and brain infarction induced by MCAO and finally improve neurological deficit. In addition, brain damage following ischemic stroke often disrupts the blood-brain barrier giving rise to an increase in paracellular permeability resulting in the extravasation of blood components into the brain and finally induces cerebral vasogenic edema [41]. Therefore, the increase in brain water content which indicated brain edema observed in this study might occur as the result of a cerebral vasogenic edema induced by brain damage and blood-brain barrier disruption. The reduction of brain edema induced by GCJ may be associated with the reduction in brain damage which in turn decreases blood-brain barrier disruption.

Our data clearly revealed that MCAO produces a significant increase in cortical expression of DNMT1 and HDAC3 in HCHF+vehicle treated rats. This finding is in agreement with the previous results obtained from many studies [42–46]. Taken all data together, we suggest that GCJ protects against brain damage and brain dysfunction in animal model ischemia stroke with metabolic syndrome condition via the suppression of DNMT1 and HDAC3 expressions in the cerebral cortex giving rise to the decrease in inflammation [42] via the reduction of IL-6 and TNF-*α* leading to an improvement of brain infarction area and brain edema together with the neurological deficit. In addition, the suppression of HDAC3 also suppresses oxidative stress which in turn decreases brain infarction area, brain edema, and neurological deficit. Due to the very high concentration of a polyphenol such as gingerol in GCJ and the neuroprotective effect against stroke of ginger [47], we suggest that the possible active ingredient may partly involve gingerol presented in GCJ. No dose-dependent effect of GCJ is observed. The possible explanation may be due to the masking effect of other ingredients and the nonlinear relationship between the observed parameters and the concentrations of GCJ.

## 5. Conclusion

**Figure 7:**
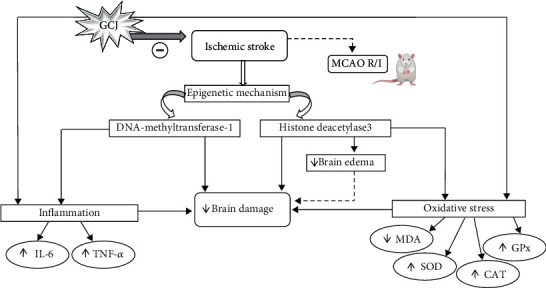
Schematic diagram of the possible underlying mechanism for the neuroprotective effect of GCJ in the animal model of cerebral ischemia in metabolic syndrome condition.

Since stroke produces a great socioeconomic burden and the current therapeutic strategy is still limited, it is very essential to prevent it [7]. This study has been focused on the noninvasive strategy for preventing the ischemic stroke with metabolic syndrome which is more severe than ischemic stroke without metabolic syndrome. This study is the first scientific evidence to prove that the reputed polyherbal recipe (GCJ) can improve brain damage and brain dysfunction induced by ischemic stroke with metabolic syndrome. Moreover, the novelty is the scientific evidence which clearly demonstrates the nutrigenomic effect of GCJ via the modulation effect on an epigenetic mechanism by suppressing the expression of DNMT1 and HDAC3 in the cerebral cortex and gives rise to the reduction of inflammation and oxidative stress resulting in an increase of cortical neuron density and the reduction in brain infarction, brain edema, and neurological deficit as shown in Figure 7. Therefore, GCJ may serve as the potential candidate food supplement for stroke protection. However, a clinical trial study to confirm this benefit is essential.

## Figures and Tables

**Table 1:** Effect of the polyherbal recipe containing ginger, Chinese date, and wood ear mushroom (GCJ) at the doses of 100, 200, and 300 mg/kg BW on oxidative stress parameters including malondialdehyde (MDA) level and the activities of superoxide dismutase (SOD), catalase (CAT), and glutathione peroxidase (GPx) in the cerebral cortex (*N* = 6/group).

Treatment group	MDA level (ng/mg protein)	SOD activity (units/mg·protein)	CAT activity (units/mg·protein)	GPx activity (unit/mg·protein)
ND + vehicle	0.29 ± 0.04	15.23 ± 0.59	38.29 ± 2.16	4.59 ± 0.41
HCHF+sham+vehicle	0.30 ± 0.06	15.41 ± 0.45	34.67 ± 4.47	3.41 ± 0.21
HCHF+MCAO+vehicle	0.68 ± 0.06^aa,bb^	6.76 ± 0.16^aaa,bbb^	15.33 ± 1.52^aaa,bbb^	2.45 ± 0.32^aa^
HCHF+MCAO+vitamin C	0.37 ± 0.04^∗^	14.74 ± 0.28^∗∗∗^	31.88 ± 2.57^∗∗^	4.27 ± 0.38^∗^
HCHF+MCAO+piracetam	0.54 ± 0.11	9.02 ± 0.06	30.91 ± 4.64^∗∗^	4.02 ± 0.22
HCHF+MCAO+GCJ100	0.36 ± 0.04^∗∗^	12.91 ± 0.49^∗∗∗^	31.61 ± 1.56^∗∗^	4.04 ± 0.38
HCHF+MCAO+GCJ200	0.35 ± 0.02^∗∗^	13.03 ± 0.35^∗∗∗^	34.21 ± 1.63^∗∗∗^	4.34 ± 0.39^∗^
HCHF+MCAO+GCJ300	0.35 ± 0.02^∗∗^	14.72 ± 1.60^∗∗∗^	31.47 ± 0.53^∗∗^	4.16 ± 0.27^∗^

Data were presented as mean ± SEM. ^aa,aaa^*p* value < 0.01 and 0.001, respectively, compared to ND+vehicle; ^bb,bbb^*p* value < 0.01 and 0.001, respectively, compared to HCHF+sham+vehicle; ^∗,∗∗,∗∗∗^*p* value < 0.05, 0.01, and 0.001, respectively, compared to HCHF+MCAO+vehicle.

## Data Availability

The data used to support the findings of this study are available from the corresponding author upon request.

## Abbreviations

BW: Body weight
CAT: Catalase
DNMT1: DNA methyltransferase 1
DTT: Dithiothreitol
EDTA: Ethylenediaminetetraacetic acid
GCJ: The polyherbal recipe containing the combined extract of ginger, Chinese date, and Jew's ear or wood ear mushroom
GPx: Glutathione peroxidase
HAT: Histone acetyltransferase
HCHF: High-carbohydrate high-fat diet
HDAC3: Histone deacetylase 3
I/R: Ischemic reperfusion injury
IL-6: Interleukin-6
MCAO: Middle cerebral artery occlusion
MDA: Malondialdehyde
MetS: Metabolic syndrome
mNSS: Modified neurological severity score systems
ND: Normal diet
SEM: Standard error of the mean
SOD: Superoxide dismutase
TNF-*α*: Tumor necrosis factor-alpha
TTC: 2,3,5-Triphenyl tetrazolium chloride.

## Supplementary Materials

Supplementary Materials Supplementary materials about dataset of the original western blot carried out in this study are attached as supplementary material.
