# Supplementary material for: Neuroprotective Effect of Polyherbal Recipe Containing Ginger, Chinese Date, and Wood Ear Mushroom against Ischemic Stroke with Metabolic Syndrome Condition via Epigenetic Modification of Inflammation and Oxidative Stress
Source: Biomed Res Int. 2022 Aug 17;2022:8940303. doi: 10.1155/2022/8940303 (PMC11401674; doi:10.1155/2022/8940303)
Supplement: Supplementary Materials — about dataset of the original western blot carried out in this study are attached as supplementary material. [file 8940303.f1.docx]

**Supplementary Data**

| **Antibodies** | **Repeat 1**  ~35  ~35 | **Repeat 2**  ~35 | **Repeat 3** |
| --- | --- | --- | --- |
| IL-6  (23 kDa) | 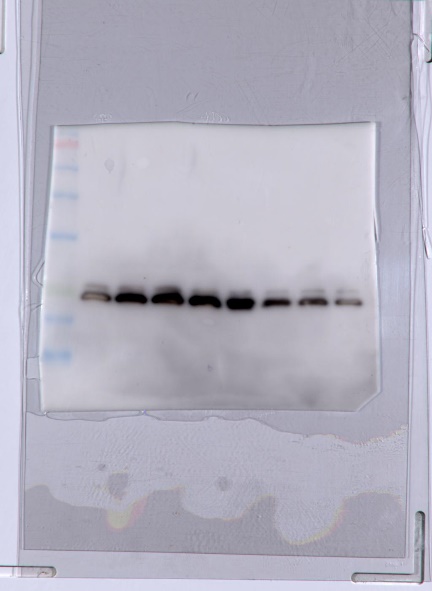  ~35  ~35  ~17  ~25  ~25  **1 2 3 4 5 6 7 8** | 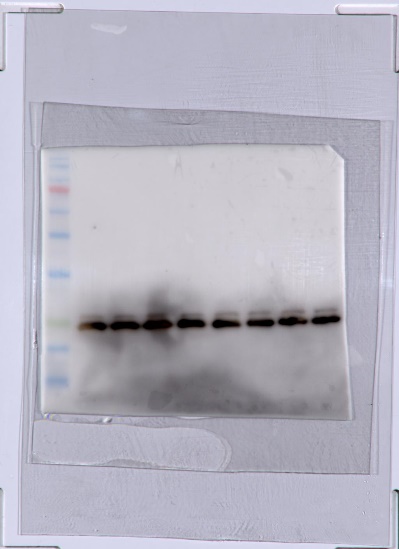  ~17  ~17  **1 2 3 4 5 6 7 8** | 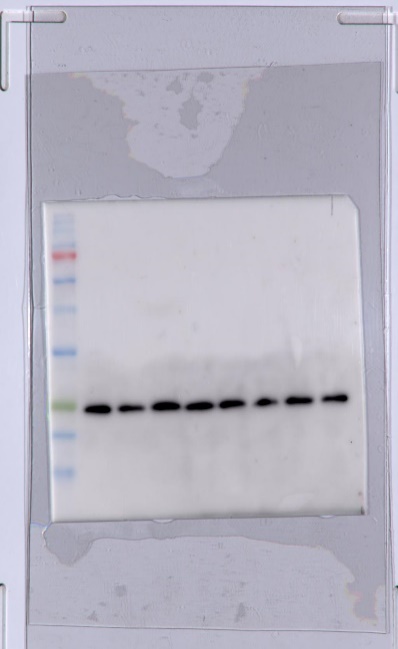  ~35  ~25  **1 2 3 4 5 6 7 8** |
| TNF-α  ~25  (25 kDa)  ~17 | 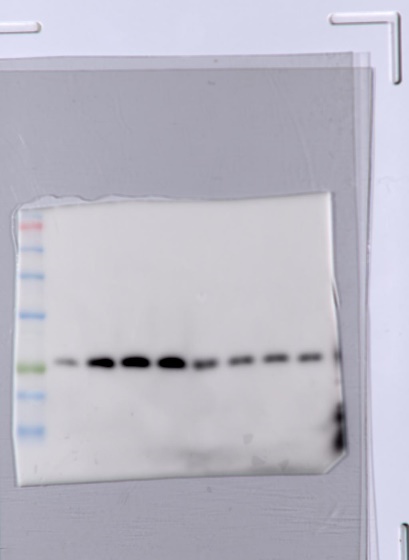  ~17  ~25  **1 2 3 4 5 6 7 8** | 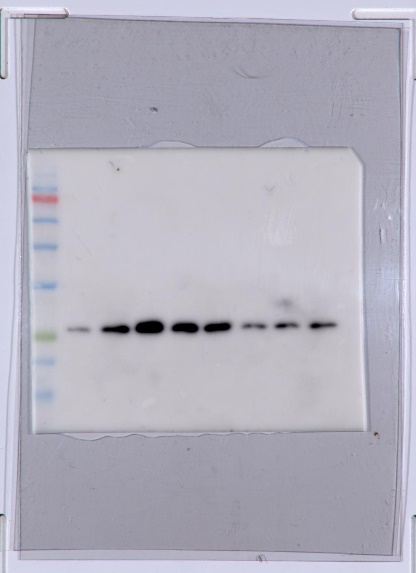  ~17  **1 2 3 4 5 6 7 8** | 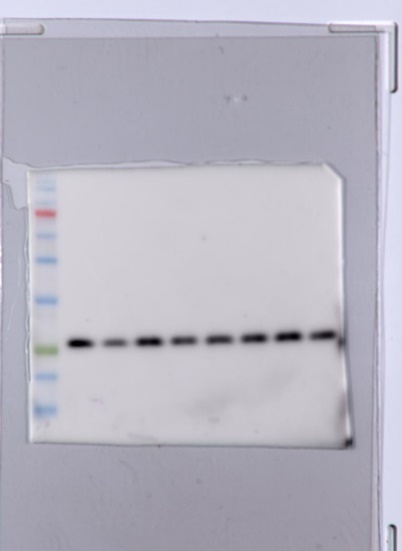  ~25  **1 2 3 4 5 6 7 8** |
| ~180  DNMT1  (183 kDa) | 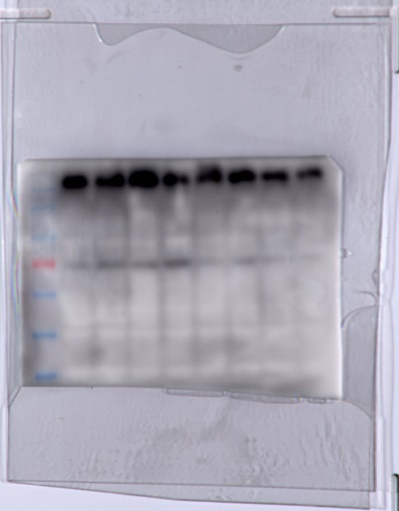  ~100  ~180  ~75  ~75  ~75  **1 2 3 4 5 6 7 8** | 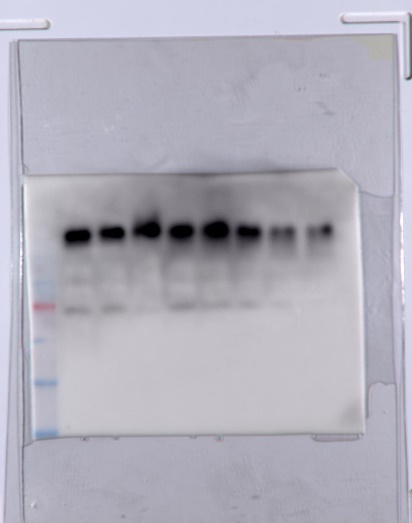  ~100  ~100  ~180  ~75  ~75  **1 2 3 4 5 6 7 8** | 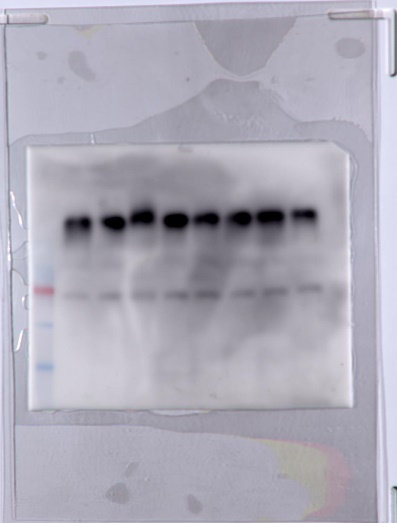  **1 2 3 4 5 6 7 8** |
| ~75  HDAC3  (50 kDa)  ~48 | 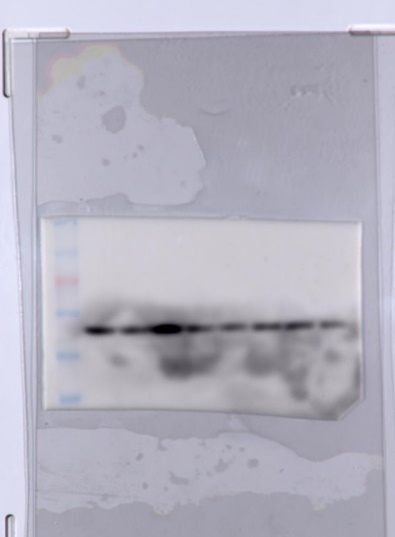  ~63  ~63  ~48  **1 2 3 4 5 6 7 8** | 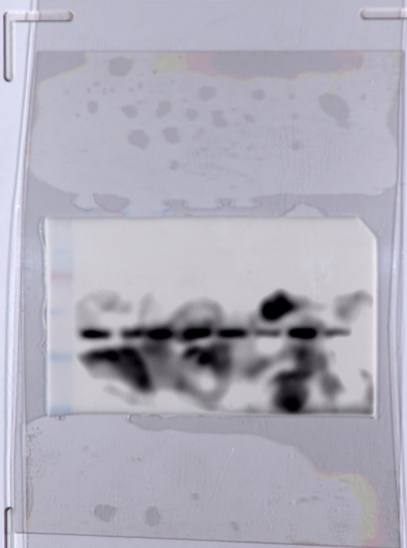  ~63  **1 2 3 4 5 6 7 8** | 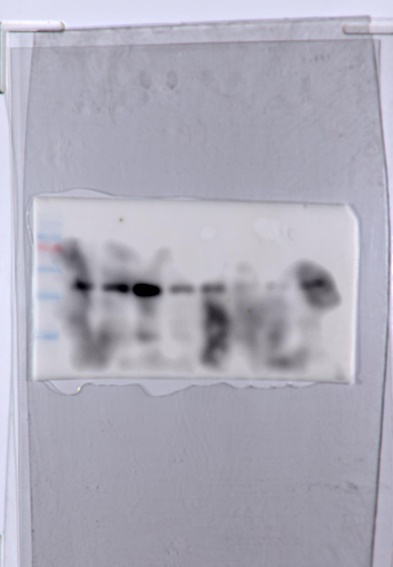  ~48  **1 2 3 4 5 6 7 8** |
| ~48  β-actin  (43 kDa)  ~25 | 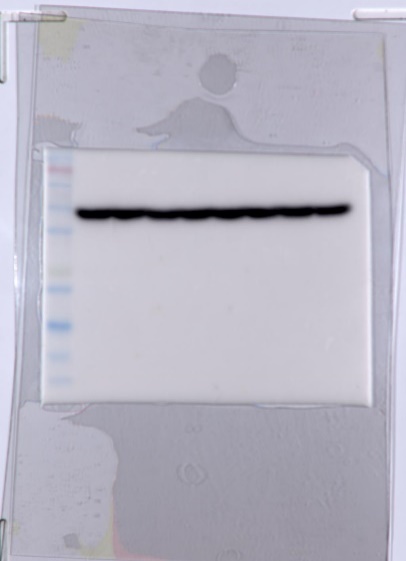  ~48  ~35  ~35  ~25  **1 2 3 4 5 6 7 8** | 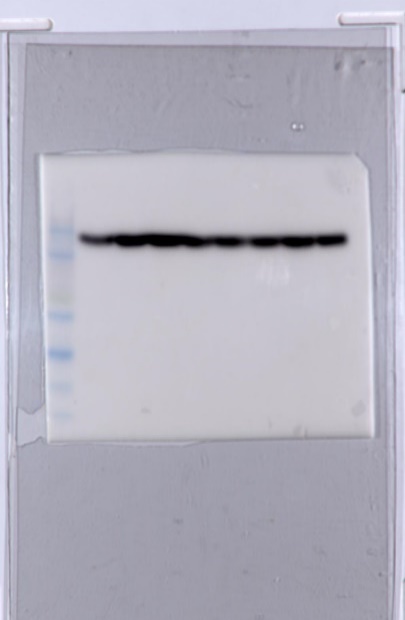  ~48  ~35  ~25  **1 2 3 4 5 6 7 8** | 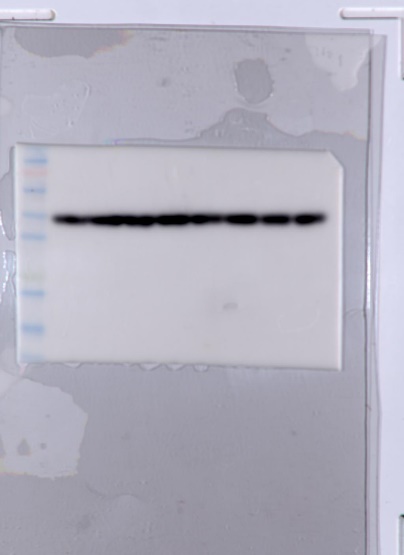  **1 2 3 4 5 6 7 8** |

Lane1; ND+Vehicle

Lane2; HCHF+Sham+Vehicle

Lane3; HCHF+MCAO+Vehicle

Lane4; HCHF+MCAO+VitaminC

Lane5; HCHF+MCAO+Piracetam

Lane6; HCHF+MCAO+GCJ100

Lane7; HCHF+MCAO+GCJ200

Lane8; HCHF+MCAO+GCJ300
